# New Investigations Concerning Epilepsy, Resulting from Certain Lesions of the Spinal Cord, and of the Rachidien Nerves

**Published:** 1870-07

**Authors:** 


### NEW INVESTIGATIONS CONCERNING EPILEPSY, RESULTING FROM CERTAIN LESIONS OF THE SPINAL CORD, AND OF THE RACHIDIEN NERVES.

By Dr. Brown-Séquard.

[Translated for the Medical Examiner.]

As space is wanting to give in this article the extensive account which the subject merits, I shall resume, briefly, some of the different points to which I wish to call the attention of my readers.

In the two preceding parts, I have examined the particulars relative to the wounds which occasion epilepsy among guinea pigs, as well as those concerning the epileptic zone, the phenomenon of the attacks, etc. I shall give a summary of the facts, commencing by some additions to the different points which have engaged me in the portions of this paper already published.

### X. Additions to the Preceding Paragraphs.

These additions have, for their especial object, the lesions which can produce epilepsy, the time of the appearance of this affection after certain lesions, the extent of the epileptic zone, the difference of excitability at divers points of this zone, finally, the spontaneous cure of epilepsy, caused by the lesion of certain nerves, or of the spinal cord.

1st. *Lesions which can produce Epilepsy.*—It is stated in the first part of this article, that I have once seen epilepsy intervene after a lesion of the rachidien bulb, in a guinea pig. From my notes in regard to this animal, I extract the following particulars, which show what are the resemblances and the differences existing between this case of epilepsy, and those where the affection is originated by the section of the sciatic nerve, or of the spinal cord.

The lesion, which consists in an extirpation of the V, of gray substance, at the calamus scriptorious, and of a part of the two restiform bodies (more on the left than the right) in the vicinity of this little, gray mass, was made upon a female guinea pig, in May, 1858. In less than two months she commenced having spontaneous attacks of epilepsy, the frequency of which became excessive. Sometimes, to my knowledge, she had as many as twelve or fifteen attacks, during a day, which evidently implies a far greater number. As a day never passed without her having at least from four to eight paroxysms, she must have had during the last fifteen months of her life, many thousand attacks. In a great number of guinea pigs which have suffered a lesion of the spinal nerve, I have seen the spontaneous attacks occur as frequently, for a few consecutive days, and even during one or two months, but never for three consecutive months.

One could provoke an attack upon her, as among all guinea pigs becoming epileptic after lesion of the nerves or spinal cord, by irritating the skin, at the angle of the lower jaw. In her, this was effected on the left side, where the lesion of the bulb was more extensive; and the convulsive movements were much more marked on the left side.* Frightening her, by a sudden motion, was frequently sufficient to occasion an attack, which was not the case among animals rendered epileptic by other lesions. Sometimes her paroxysms, either spontaneous or provoked, were followed by unmistakable coma. This, I have never observed in other guinea pigs, whatever may have been the cause of their epilepsy.

There is, then, respecting the two characteristic traits, which consist in the production of epilepsy, and in that of a cutaneous,

* The animal had a wheeling to the left (like a movement in horsemanship) for many days after the lesion of the rachidien bulb; this tendency to wheeling continued throughout its life. When the animal was frightened, without causing an attack of epilepsy, the body frequently would curve to the left, until the nose almost touched the sacrum. Hyperæsthæsis and an elevation of temperature existed in the left limbs. There were many slightly sensitive spots on the face, especially on the left side. The eyes and ears, on the left side particularly, were the seat of alterations, which I shall describe hereafter, in a work upon the influence of lesions of the restiform bodies upon nutrition, in these parts.

epileptic zone, a perfect similitude between the effects of lesion of the bulb, in the case just described, and those observed after certain lesions of the spinal centre, and of its nerves, among guinea pigs.

*Time of the Appearance of Epilepsy.*—I have stated in the preceding number, that in two cases, the first reflexive, convulsive phenomena have shown themselves the third day after the section of the sciatic nerve. I have just seen some phenomena of this kind, upon irritating the epileptic zone only forty-five hours after this injury. But these first symptoms may also be much retarded in their appearance, occurring twenty days, and in one instance, twenty-two days after the lesion.

Previous to the 25th of April last, the earliest complete attack following the section of the sciatic, or of the internal popliteal nerve, aside from the one just mentioned, has occurred on the eighth day. Since then, I have observed the first attack appear in one instance on the sixth, and in another on the seventh day. The earliest complete attack I have observed, after the section of the posterior roots of the spinal nerves forming the sciatic, and that of the roots of the last four or five dorsal pairs, has been eight days after the first of these lesions, and thirteen days after the second.

The early development of the first reflex phenomena bears no fixed relation with that of the first complete attack. For example, I have seen reflex, convulsive movements manifested in four days after the section of the sciatic, in an animal that had its first attack after twenty-one days, and, on the contrary, I have produced the first completed paroxysm, the fifteenth day after this lesion, in a guinea pig, which had the first convulsive symtoms only three days previously. Many similar experiments have induced me to consider it probable that the frequently repeated irritation of the skin of the neck and face have developed the epileptic faculty there sooner than it would have naturally manifested itself after the lesions which produce epilepsy.

*Extent of the Epileptic Zone.*—In guinea pigs that have suffered a section of the lateral half of the spinal nerve, in the region of the last dorsal or first lumbar vertebræ, the epileptic zone may extend to other portions than those whose limits I described in the first part of this paper. After a certain time, it almost always extends from, the omoplate to the middle line of the back, and occupies a narrow band (about one centimetre in width) along this line to the sixth, seventh, or eighth dorsal vertebræ, on the side of the lesion. I observed repeatedly that pinching this little band of skin would provoke an attack, but noticing that before the appearance of the convulsion the animal would turn its head towards the pinched part, I believed for a long time that the friction occasioned by this movement sufficed to explain the paroxysm. But I have been convinced recently by the most positive evidence that this irritating the skin of one of the vertebral grooves is really the direct cause of the attack; for in some cases it has appeared the moment I pinched the skin, and in other instances, where I could, without irritating the epileptic zone of the head and neck, prevent any turning of the head toward the injured side, I have witnessed the convulsions, after pinching this band of skin alluded to.

I will say in addition, that when epilepsy has continued a long time, after a lateral hemisection of the spinal cord, the skin covering the vertebral groove, especially in the vicinity of the omoplate, may possess the epileptic faculty in a much greater degree than that of the face.

It seems certain, then, that after a section of the lateral half of the spinal nerve, at the level of the last dorsal, or the first lumbar vertebræ, the epileptic zone includes generally, if not invariably, in addition to a portion of the skin of the face or neck (see fig. 6, pl. V.), a narrow band of skin, extending over one of the vertebral grooves, from the neck unto the sixth, seventh, or eighth dorsal vertebra.

From long continued observations, but more particularly from those of recent date, it appears that the extent of the epileptic zone along the back is diminished, when the lateral hemisection of the spinal nerve approaches the caudal extremity. For example, in a guinea pig now under my notice, the section was made at the level of the fifth lumbar vertebra, and the epileptic zone is limited to the face and a small portion of the neck, not extending to the omoplate, while in another, having the lateral hemisection of this nervous centre at the level of the eighth dorsal vertebra, the zone extends unto the cicatrix.

*Degree of Excitability in different parts of Epileptic Zone.*— In double epilepsy, which is caused by one lesion on the right, and another on the left, consisting either in a section of the two sciatic nerves, or of a traverse section of the posterior half, or the entire thickness of the spinal nerve, or finally, of the section of the sciatic nerve on one side, and a lateral hemisection of the nervous centres on the other, there is generally more excitability in the portion of skin covering the spinal processes of the first six or seven dorsal vertebræ, than elsewhere. Nevertheless, the point of the epileptic zone, possessing the greatest power of engendering an attack, varies considerably in cases of transverse section, either partial or complete, of the spinal nerve, or even in cases of section of one or both sciatic nerves. In this respect, the dissimilarity is not entirely attributable to the kind of lesion, as a difference is noticed in animals that have suffered the same lesion, and even in the same animal, at various times. Meanwhile, the points which are generally the most excitable, are the little portions of skin covering the angle of the lower jaw, the anterior and superior borders of the omoplate, the side of the spinal processes of the fourth, fifth, and sixth dorsal vertebra, and finally, the skin of the cheek, below the eye.

*Spontaneous Cure of Epilepsy, produced by the Section of certain parts of the Spinal Cord, or of certain Nerves.*—I can repeat what I have previously said, that I have never seen a spontaneous cure of a guinea pig, rendered epileptic by a lesion of the spinal cord. I can also affirm the same of animals becoming epileptic after the *section* of the sciatic nerve; of the internal popliteal; of the posterior roots of the sciatic nerve; or of those of the last dorsal. But I have seen a case of spontaneous cure in a guinea pig that had suffered a mechanical *irritation* of the sciatic nerve. At present, I only mention this fact, but I shall show hereafter that it bears a very important relation to the theory of epilepsy.

When the lesions of the nerves, or of the spinal centre, are incapable of producing complete epilepsy, but at the same time sufficient to occasion some reflex convulsive movements, after irritation of the epileptic zone, the spontaneous cure generally takes place within six or eight weeks. If I had not killed a number of animals before their cure, I might have been able to say this was always the case.

I wish to remark that here only the *spontaneous* cure is mentioned. In another article I shall call attention to curability under different methods of treatment.

XI. Production of Epileptic Attacks, after the extirpation of the Cerebrum, the Cerebellum, and other parts of the Encephalon.

Long since, I have demonstrated in my course the important fact that, in guinea pigs having suffered a partial or complete section of the spinal nerve, an attack can still be produced, after removing a considerable portion of the encephalon.

While maintaining life by pulmonary insufflation, in epileptic guinea pigs, from whom I have removed the cerebrum, the cerebellum, and a great portion of the annular protuberance, I have succeeded in producing a number of paroxysms, entirely similar to those taking place in these animals before the mutilation of the encephalon. The attack can be produced by pinching not only the skin of the neck and the vertebral grooves, but also the portions of the epileptic zone receiving the ramifications of the auriculo temporal and of the suborbital nerves.

* This animal is the only one which I have known to survive complete attacks of epilepsy, after an irritation of the sciatic nerve. I compressed the nerve strongly with a pair of forceps, on the 4th of May. This irritation produced anesthæsia and paralysis of the limb. On the 13th of May, the reflex, convulsive movements were already strong. The 15th, a violent, *incomplete* attack took place. The first complete attack occurred on the 20th. During the first days of June, I could only induce some reflex, convulsive movement, which declined in intensity from day to day, and on the 10th of June, there was no longer any trace of epileptiform affection. At the same time that the amelioriation of the convulsive phenomena began, the voluntary motion reappeared in the foot, whose nerve had been injured. To-day (June 22), there is no trace of any morbid state whatever in the animal.

I have long ago ascertained, among animals epileptic or not, when the loss of blood has not been profuse, after the extirpation of a great part of the encephalon, that pulmonary insufflation augments the reflex faculty of the rachidien bulb and of the spinal cord, as well as the excitability of the nerves and muscles. I have not been surprised, then, in finding that when insufflation has been practiced for eight, ten, or twelve minutes, upon epileptic guinea pigs, the facility of provoking attacks by irritation of the epileptic zone is augmented materially.

In one case I was able yet to produce paroxysms after the extirpation of all the encephalon, except the rachidien bulb, and, in another animal, I even caused a very severe convulsion, after at once cutting across the rachidien bulb, immediately below the origin of the pneumo-gastric nerves, having previously maintained pulmonary insufflation for ten minutes. It is not necessary to say that exciting the portions of the epileptic zone, animated by the trigeminal, proved ineffectual, and that it was irritation of the skin of the neck, and of the posterior part of the back, which occasioned the attack. The animal became epileptic in consequence of a complete transverse section of the spinal cord, at the level of the tenth dorsal vertebra.

My colleague Vulpian was the first to make the experiment showing that attacks of epilepsy can still occur, after the removal of a large part of the encephalon, in animals epileptic in consequence of the section of the sciatic nerve.*

I have performed it many times since, and always with the same results that I have mentioned above. I will remark, in reference to the experience of my colleague, that if he has believed an attack could be produced by any irritation whatever, instead of the irritation of a certain zone only, it is because, although not recognizing the epileptic influence of this zone, he held the animal by the neck, while he irritated it elsewhere. The guinea pig struggling, rubbed the neck or the face against the hand which retained it, and the epileptic zone thus received the irritation which occasioned the convulsion.†

* See *Archives de Physiol. norm*, etc., vol. II, 1869, page 297.

† See note, page 433, of the second part of this paper, *Archives de Physiology*, May, 1869.

Within a recent period, a German physiologist,* Nothnagel, has published a work designed to show that the annular protuberance is the only nervous centre capable of producing epileptiform convulsions. In another paper, discussing the theory of spontaneous and provoked epilepsy, I shall demonstrate that this opinion is erroneous, and that other portions of the nerve centres, as well as the protuberance, can give rise to epileptic paroxysms.

In a second paper, I shall present the results I have obtained; first, upon the means of transmitting the irritation of the spinal nerves, or of the spinal cord to the encephalous, and to the skin of the epileptic zone, in order to produce there the alterations of nutrition, which occasion epilepsy: secondly, upon the condition of the sciatic and other nerves, which produce epilepsy; thirdly, upon the difference between many animals† and man, in respect to the production of epilepsy by irritation of certain nerves, or of the spinal centre; fourthly and finally, upon other different questions concerning the pathological physiology of epilepsy, and of the convulsive epileptiform movements.

* Virchow's *Archiv. fur Pathol. Anat.*, 1868, Vol. XLIV, Page 1.

† Epilepsy, with the existence of the epileptic zone, in cases of lesion of the sciatic nerve, has been found to exist not only in guinea pigs, but also in man, by Dr. G. Dieulafay, in the cat, by Mr. Trasbot, and in the rabbit, by my own experiments.

